# Pharmacokinetics, Tissue Distribution and Excretion of Isoalantolactone and Alantolactone in Rats after Oral Administration of Radix Inulae Extract

**DOI:** 10.3390/molecules20057719

**Published:** 2015-04-28

**Authors:** Renjie Xu, Guisheng Zhou, Ying Peng, Mengyue Wang, Xiaobo Li

**Affiliations:** School of Pharmacy, Shanghai Jiao Tong University, No.800 Dongchuan Road, Minhang District, Shanghai 200240, China; E-Mails: xu8522333@163.com (R.X.); zhouguisheng1@126.com (G.Z.); ypeng@sjtu.edu.cn (Y.P.); mywang@sjtu.edu.cn (M.W.)

**Keywords:** Radix Inulae, isoalantolactone, alantolactone, pharmacokinetics, tissue distribution, excretion

## Abstract

Radix Inulae is endemic to China and has been used in traditional medicine to treat upper body pain, emesis and diarrhoea, and to eliminate parasites. Here, an UPLC-MS/MS method was developed and applied to study the pharmacokinetics, distribution and excretion of isoalantolactone and alantolactone, which are two main active sesquiterpene lactones in Radix Inulae, in Sprague-Dawley rats following oral administration of total Radix Inulae extract. Isoalantolactone, alantolactone and osthole (internal standard) were prepared using acetonitrile precipitation, and the separation of isoalantolactone and alantolactone was achieved by isocratic elution using water (containing 0.1% formic acid) and acetonitrile as the mobile phase using a ZORBAX Eclipse Plus C_18_ column. The total run time was 6.4 min. The present study showed poor absorption of isoalantolactone and alantolactone *in vivo*. The apparent C_max_, T_max_, T_1/2_ and total exposure (AUC_0–12h_) in rat plasma were 37.8 ng/mL, 120 min, 351.7 min and 6112.3 ng-min/mL for isoalantolactone and 25.9 ng/mL, 90 min, 321.0 min and 4918.9 ng-min/mL for alantolactone, respectively. It was shown that the highest concentration was achieved in the small intestine and feces clearance was shown to be the dominant elimination pathway of the lactones.

## 1. Introduction

**Figure 1:**
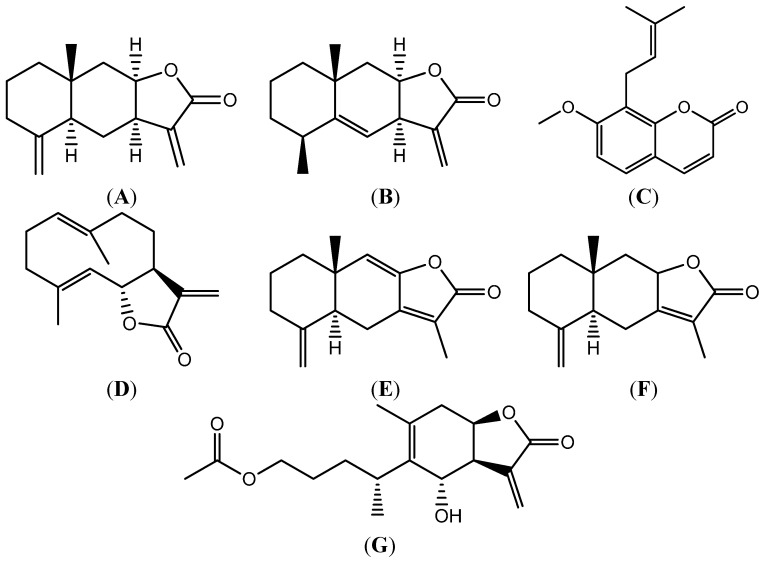
Structures of isoalantolactone (**A**), alantolactone (**B**), osthole (**C**), costunolide (**D**), atractylenolide I (**E**), atractylenolide II (**F**) and 1-acetoxy-6α-hydroxyeriolanolide (**G**).

Radix Inulae, a commonly used folk medicine called Tu-Mu-Xiang or Zang-Mu-Xiang in China, is the root of *Inula helenium L.* or *I. racemosa Hook. f.* [1,2], and belongs to the Asteracea family. In TCM therapy, Radix Inulae is used to treat upper body pain, emesis and diarrhoea, and to eliminate parasites [3]. In our previous studies Radix Inulae extract was also shown to be useful in the therapy of Irritable Bowel Syndrome (IBS). Isoalantolactone (Figure 1A) and alantolactone (Figure 1B) are the main active substances in the chloroform extract from Radix Inulae [4]. Isoalantolactone and alantolactone has been demonstrated to have a wide range of activities *in vivo* and *in vitro* [5], including anti-microbial [6,7,8], anti-trypanosomal [9], and anti-inflammatory activities [10] and they had strong anti-tumor effects on several cancer cell lines. It was reported that alantolactone had potential activity against glioblastoma cells [11], RKO cells [12], triple-negative breast cancer MDA-MB-231 cells [13] and lung squamous cancer SK-MES-1 cells [14] while isoalantolactone was proved to induce apoptosis in pancreatic carcinoma PANC-1 cells [15], chronic myelogenous leukemia [16], K562/A02 cells [17] and SGC-7901 cells [18].

Although the biological activity of Radix Inulae and the two lactones has been observed, many of their key characteristics for drug evaluation are yet unknown, such as the pharmacokinetic parameters, the *in vivo* preference in tissue distribution and excretion after oral administration [19,20,21]. Some studies concerning the pharmacokinetics of isoalantolactone and alantolactone have been published, but only intravenous injection condition was evaluated [22,23]. Thus, additional research is needed since Radix Inulae was mainly administered via oral administration instead of via intravenous injection.

Meanwhile, increasing evidence has demonstrated that LC-MS/MS was now the best method for such studies [24] with a much lower limit of quantification (LOQ) than other measurements like high-performance liquid chromatography (HPLC) with ultraviolet (UV) detection [25]. In addition, chromatographic performance has been greatly improved by the availability of ultra-performance liquid chromatography (UPLC) compared to conventional HPLC methods [26]. The systematic and comprehensive knowledge on Radix Inulae is not only required for understanding its pharmacological effects and protective mechanisms, but can also provide scientific evidence to support the clinical use of Radix Inulae. For these purposes, in this work we examined and compared the pharmacokinetic, tissue distribution and excretion in rats after oral administration of the Radix Inulae using a validated UPLC-MS/MS method.

## 2. Results and Discussion

### 2.1. Optimization of Separation Conditions

A simultaneous determination of isoalantolactone and alantolactone in rat plasma after intravenous injection was reported in a recent study by Guo *et al*. [22]. In the study, HPLC was used for the separation of the two lactones. Total analysis time of this method is very long, almost 19 min, and the retention time of IS and lactones was quite different (6.4 min *versus* 12.4 min). In another study, a sensitive and rapid UPLC-MS/MS method was developed for determination of the two compounds in rat plasma separately, with a total analysis time of less than 3 min, but it failed to determine isoalantolactone and alantolactone simultaneously [23]. In the present study, an isocratic elution using water (containing 0.1% formic acid) and acetonitrile as the mobile phase was established for the separation of isoalantolactone and alantolactone. The total analysis time was only 6.4 min, while isoalantolactone, alantolactone and IS were eluted at approximately 2.89, 3.13 and 2.21 min, respectively. The new method was not only applied to detect isoalantolactone and alantolactone simultaneously in rat plasma but also in the analysis of the two lactones in organs, bile, urine and feces matrix.

### 2.2. Method Validation

**Figure 2:**
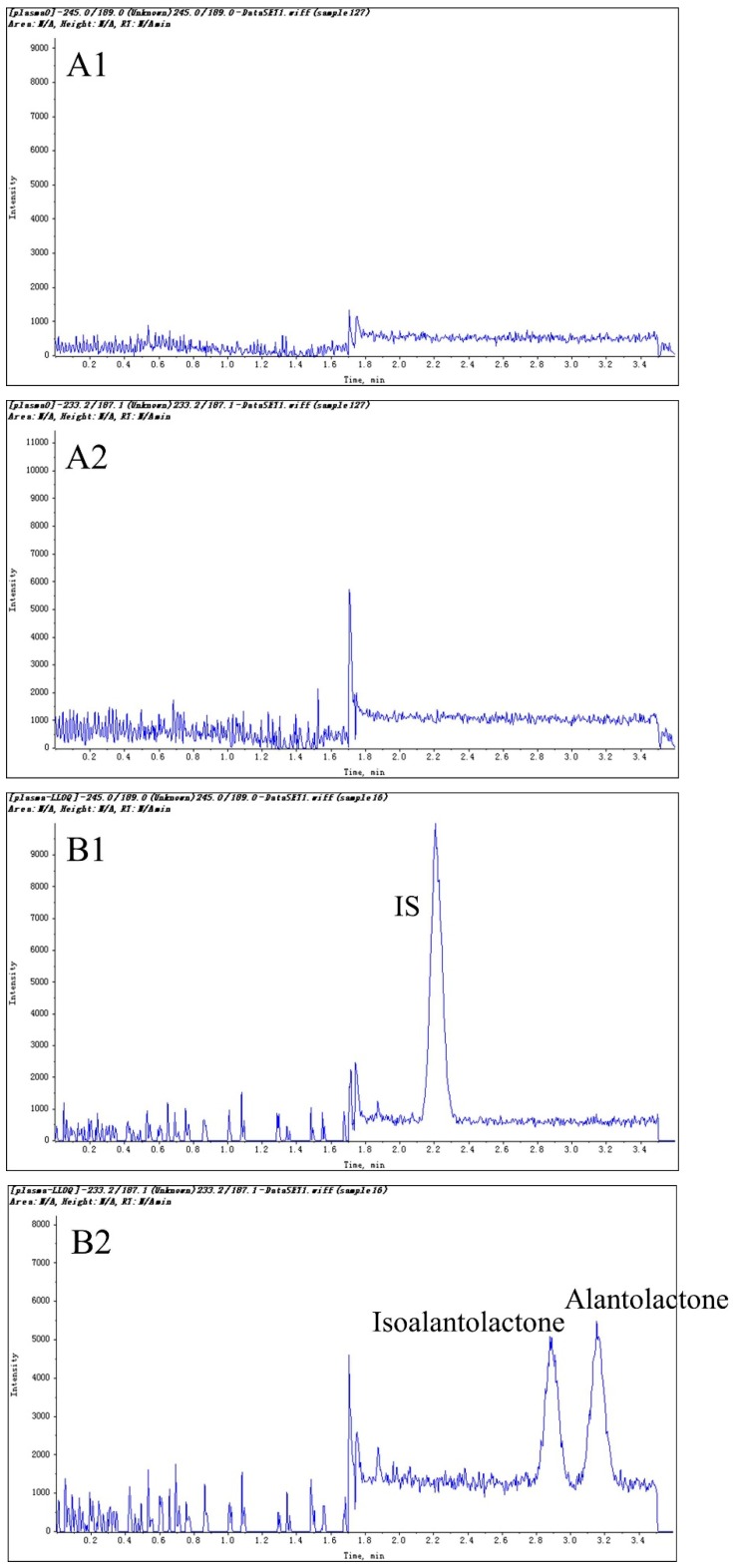

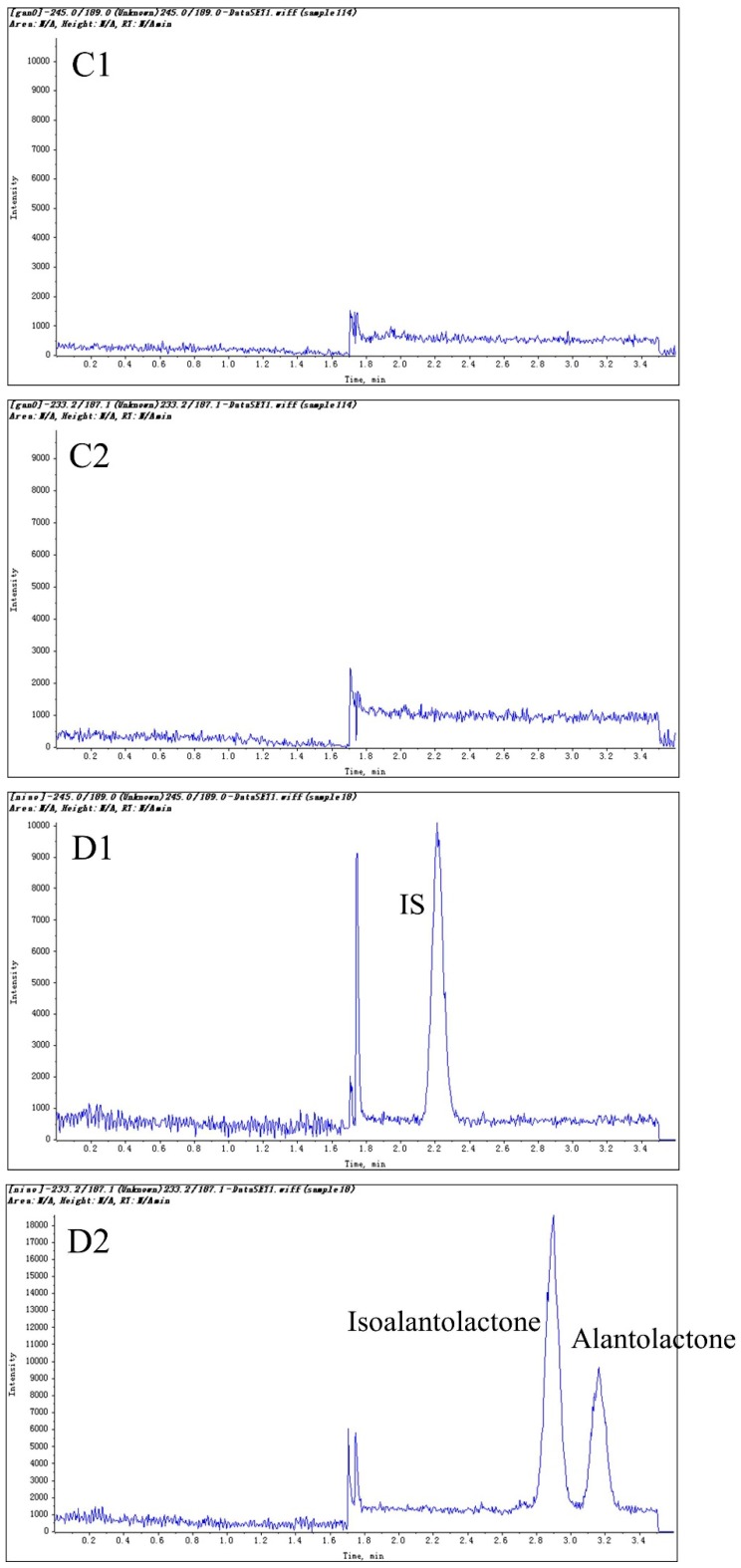

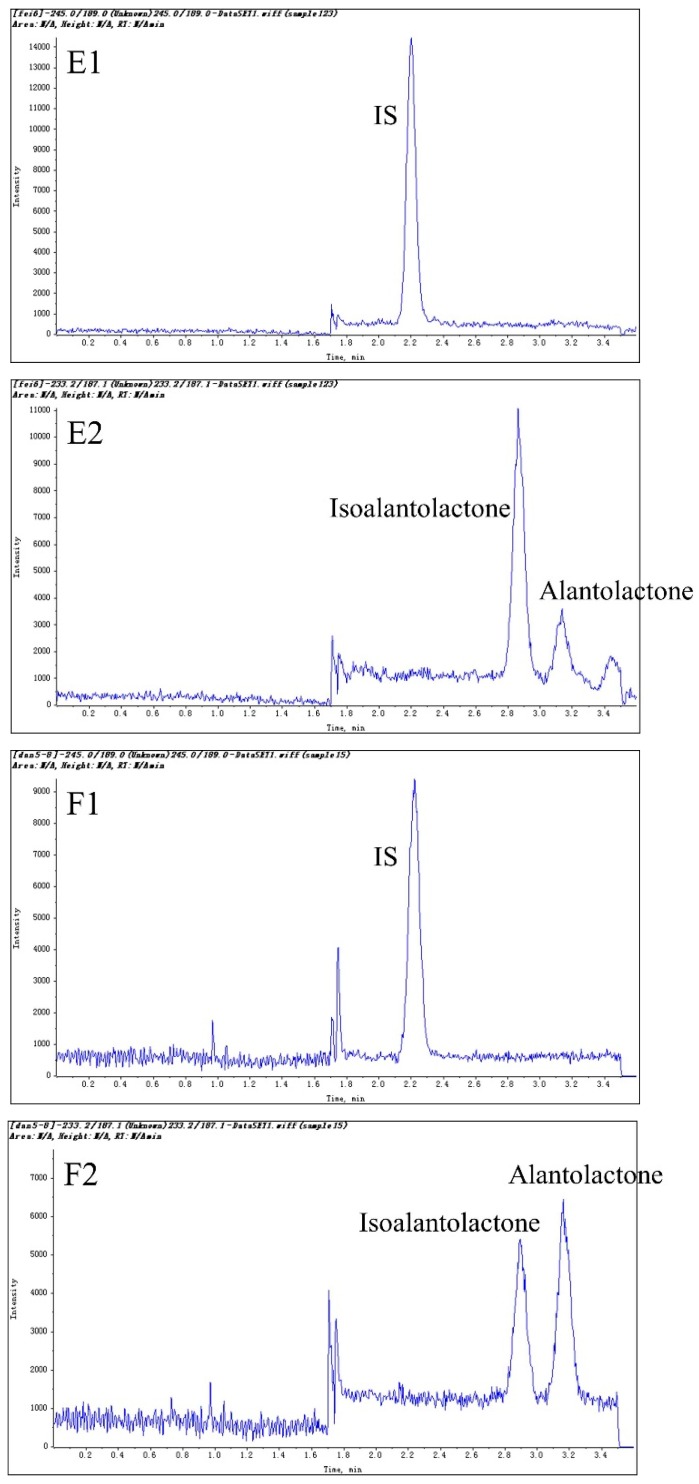
Representative multiple reaction monitoring chromatograms of (**A**) a blank plasma sample of rats; (**B**) a plasma spiked with 2 ng/mL isoalantolactone, 4 ng/mL isoalantolactone (LLOQ) and IS (0.5 ng/mL); (**C**) a blank rat liver sample of rats; (**D**) a blank rat urine sample spiked with isoalantolactone (25 ng/mL), alantolactone (25 ng/mL) and IS (0.5 ng/mL); (**E**) a rat lung sample with IS at 12 h after oral administration of Radix Inulae extract (containing 7.06 ng/mL isoalantolactone and alantolactone with concentration under LLOQ) and (**F**) a rat bile sample with IS between 5 and 8 h after oral administration of Radix Inulae extract (containing 507.65 ng/mL isoalantolactone and 899.85 ng/mL alantolactone).

Representative chromatograms of blank plasma, blank liver, blank plasma spiked with lactones at LLOQ and IS, blank urine spiked with lactones and IS, a lung sample after oral administration of Radix Inulae extract spiked with IS and a bile sample after oral administration of Radix Inulae extract spiked with IS are shown in Figure 2. No detectable interfering peaks were found with the retention time close to that of lactones and internal standard as shown in Figure 2.

The calibration curves were generated by plotting the peak-area ratios of lactones to IS *versus* the nominal concentrations in the standard biological samples. All calibration curves for both isoalantolactone and alantolactone showed good linearity over the concentration range studied (all correlation coefficients > 0.9900) as shown in Table 1. 

**Table 1:** Standard curves, correlation coefficients and linear ranges of lactones in tissue samples (*n* = 5).

Compound	Calibration Curves (y = ax + b)		Linear Range (ng/mL)	Correlation Coefficients (r^2^)
	a	b		
Plasma				
Isoalantolactone	0.1005 ± 0.01523 ^a^	0.02750 ± 0.004353	2–100	0.9986 ± 0.002343
Alantolactone	0.05870 ± 0.006154	−0.03411 ± 0.007921	4–100	0.9992 ± 0.001666
Heart				
Isoalantolactone	0.1470 ± 0.01211	−0.2358 ± 0.03101	2–100	0.9951 ± 0.001135
Alantolactone	0.03920 ± 0.007460	−0.0885 ± 0.01510	4–100	0.9977 ± 0.001332
Spleen				
Isoalantolactone	0.1221 ± 0.02341	−0.0892 ± 0.02549	2–100	0.9976 ± 0.002145
Alantolactone	0.04770 ± 0.003254	−0.1555 ± 0.02931	4–100	0.9997 ± 0.001819
Liver				
Isoalantolactone	0.1025 ± 0.008433	0.1250 ± 0.02631	2–400	0.9976 ± 0.002331
Alantolactone	0.03940 ± 0.005400	−0.051 ± 0.008120	4–400	0.9997 ± 0.001510
Kidney				
Isoalantolactone	0.1249 ± 0.008918	−0.0381 ± 0.005122	2–100	0.9996 ± 0.001721
Alantolactone	0.0550 ± 0.006371	0.0553 ± 0.008688	4–100	0.9954 ± 0.002435
Lung				
Isoalantolactone	0.1249 ± 0.01511	−0.0381 ± 0.009131`	2–100	0.9979 ± 0.002063
Alantolactone	0.06310 ± 0.004242	0.0150 ± 0.006626	4–100	0.9906 ± 0.002023
Stomach				
Isoalantolactone	0.1192 ± 0.02131	−0.0928 ± 0.02325	2–200	0.9949 ± 0.001754
Alantolactone	0.0550 ± 0.004276	0.0553 ± 0.009178	4–200	0.9934 ± 0.002197
Small intestine (colon)				
Isoalantolactone	0.1156 ± 0.02943	0.0828 ± 0.01054	2–200	0.9957 ± 0.001845
Alantolactone	0.04211 ± 0.002544	−0.0234 ± 0.004461	4–200	0.9911 ± 0.001731
Urine				
Isoalantolactone	0.1148 ± 0.009552	0.6868 ± 0.09312	2–400	0.9976 ± 0.002236
Alantolactone	0.05501 ± 0.003311	0.5318 ± 0.08411	4–400	0.9993 ± 0.001943
Bile				
Isoalantolactone	0.001334 ± 0.03143	0.0212 ± 0.005646	2 × 10^3^–4 × 10^5^	0.9961 ± 0.002821
Alantolactone	0.0006733 ± 0.009228	−0.0969 ± 0.01834	4 × 10^3^–4 × 10^5^	0.9975 ± 0.002967
Feces				
Isoalantolactone	0.001435 ± 0.01934	0.0378 ± 0.006563	2 × 10^3^–5 × 10^5^	0.9991 ± 0.003498
Alantolactone	0.0007112 ± 0.004001	0.067 ± 0.009100	2 × 10^3^–5 × 10^5^	0.9989 ± 0.003530

^a^ Mean ± SD.

The LLOQ in collected samples was 2 ng/mL for isoalantolactone and 4 ng/mL for alantolactone. Assay accuracy at low, medium, and high QCs was −11.92–13.20 for isoalantolactone and −9.39–9.45 (*n* = 6) for alantolactone. Precision (RSD) was 2.23%–9.83% for isoalantolactone and 3.09%–9.56% for alantolactone. By determining the absolute peak areas of lactones at all the three QC levels in six samples, the matrix effect and recovery of the method were evaluated, including standards, standards spiked prior to the extraction, and standards spiked after extraction in six samples. Matrix effect was calculated by dividing the peak area of isoalantolactone and alantolactone in samples spiked post-extraction by that of the acetonitrile dissolved samples. The extraction recovery was calculated by dividing the peak area of standards spiked prior to the extraction by that of the isoalantolactone and alantolactone spiked post-extracted sample. Extraction efficiency was ranged from 80.57% to 95.38% at various QC concentrations for the two compounds (*n* = 6), and no matrix effect was observed for any compound in the QCs (*n* = 6, Supplementary Material). The stability of isoalantolactone and alantolactone in rat plasma has been investigated in the previous study [23] and the result showed that the two lactones had acceptable stabilities after three freeze and thaw cycles, and were stable at room temperature (20 °C) for 4 h, at −80 °C for 20 days, or in the sampler for 24 h. Dilution effect was investigated to ensure that dilution with blank matrix would not affect the final concentration. Isoalantolactone and alantolactone spiked rat plasma samples were prepared at 25 μg/mL (for both lactones, respectively) and then diluted with pooled rat biological samples at dilution factors of 100 and 500 in six replicates before analysis. The results from six replicates showed satisfying accuracy and reproducibility, and therefore indicated that such a method using acetonitrile precipitation could be applied to the entire scope of concentrations tested in this study.

### 2.3. In Vivo Pharmacokinetic Studies

**Figure 3:**
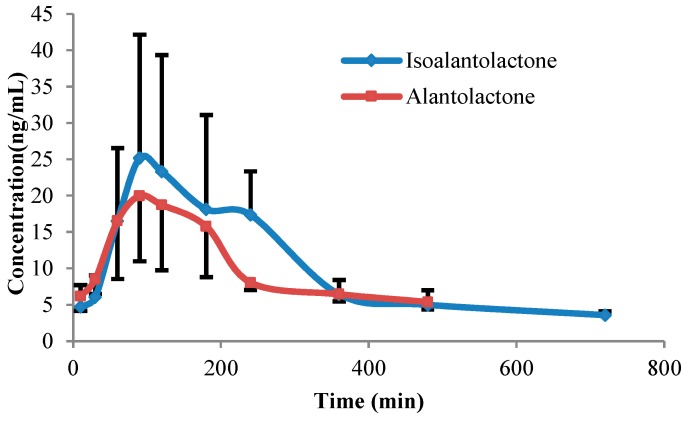
Plasma concentration-time profiles of isoalantolactone and alantolactone following oral administration in rats at doses of 90 mg/kg Radix Inulae extract (*n =* 6).

Drug and statistics software Kenitica 4.4.1 (Thermo Electron Corporation, Waltham, MA, USA) was used to analyze plasma lactones concentration over time in each experimental rat. The plasma concentration-time profiles of lactones in rats following oral administration at doses of 90 mg/kg are shown in Figure 3 and the corresponding pharmacokinetic parameters are summarized in Table 2. As shown in Table 2, the plasma concentration of isoalantolactone and alantolactone reached the maximum concentration at 37.8 ± 15.3 and 25.9 ± 9.3 ng/mL, respectively. The pharmacokinetic parameters were determined and the apparent T_max_ was 120 ± 50.2 min for isoalantolactone and 90 ± 26.8 min for alantolactone. The total exposure (AUC) was 6112.3 ± 2045.2 min-ng/mL for isoalantolactone and 4918.9 ± 755.8 min-ng/mL for alantolactone.

**Table 2:** Pharmacokinetic parameters of lactones in rats.

Parameter	Oral Administration (90 mg/kg)	
	Isoalantolactone	Alantolactone
T_1/2_ (min)	351.7 ± 158.7 ^a^	321.0 ± 222.4
MRT (min)	501.2 ± 197.8	479.2 ± 278.4
C_max_ (ng/mL)	37.8 ± 15.3	25.9 ± 9.3
T_max_ (min)	120 ± 50.2	90 ± 26.8
AUC_0–12_ (ng-min/mL)	6112.3 ± 2045.2	4918.9 ± 755.8
AUC_12–∞_ (ng-min/mL)	1248. 3 ± 488.7	1151.5 ± 397.0
AUC_0–12_/AUC_0–∞_	0.83	0.81

^a^ Mean ± SD.

These results are similar to the findings from previously published studies on other sesquiterpene lactones. For example, costunolide (Figure 1D), atractylenolide I (Figure 1E) and atractylenolide II (Figure 1F) all showed relatively low C_max_ (less than 25 ng/mL) in rats after oral dosage of 10 mg/kg or higher. (19.84 ng/mL, 7.99 ng/mL, non-detectable, respectively) [27,28]. Even 1-acetoxy-6α-hydroxyeriolanolide, measuring the highest C_max_ (609.03 ± 165.43 ng/mL) among all sesquiterpene lactones analyzed so far in the literature, could not reach a concentration of 1 μg/mL after oral administration [29]. Consistently, none of the above lactones exhibited a high volume of AUC(0–t) [27,28,29]. Given these facts, there seems to be a trend that the sesquiterpene lactones family are readily available after oral administration, though a more thorough study on absolute bioavailability would further confirm this conclusion.

### 2.4. Tissue Distribution Studies

**Figure 4:**
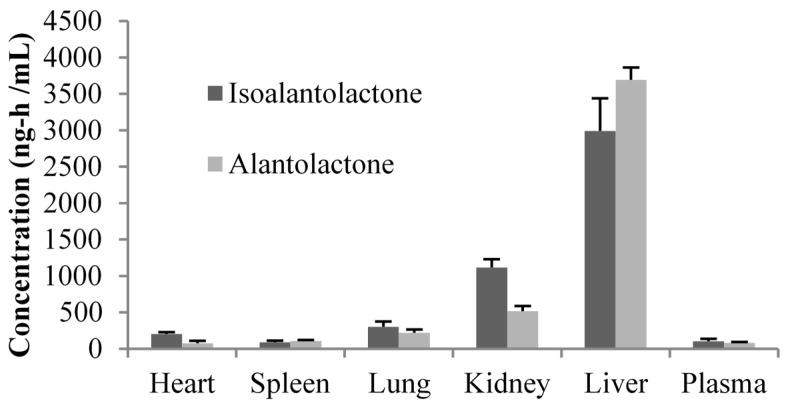
The AUC_(0-48h)_ of isoalantolactone and alantolactone in tissues following oral administration at a single dose of 90 mg/kg Radix Inulae extract (*n* = 6).

The tissue distribution of lactones after oral administration of 90 mg/kg Radix Inulae extract in rats at 1, 3, 5, 8, 12, 24 and 48 h is presented in Table 3. The results indicated that isoalantolactone and alantolactone were widely distributed throughout the body. Three hours after administration of Radix Inulae extract to rats, peak concentration of both isoalantolactone and alantolactone was observed in all collected tissues except stomach and colon. It seemed that isoalantolactone and alantolactone were mainly distributed to the gastrointestinal tract during the first 1–3 h after administration, which was not unexpected since the drug was administered orally. The ranking of maximum concentrations among all non-gut tissues was as followed: liver > kidney > lung ≈ heart > plasma > spleen for isoalantolactone and liver > kidney > lung > spleen > plasma ≈ heart for alantolactone. Distribution of lactones in the liver (AUC0-48) was more pronounced than in any other tissues but the gastrointestinal tract (Figure 4). Hence, it was hypothesized that excretion of both isoalantolactone and alantolactone occurred primarily via the liver rather than urine and discharged through the bile. The *in vivo* process of drug is so complex and further researches are needed to explain the difference in tissue distribution between the isomers.

**Table 3:** Isoalantolactone and alantolactone concentration (ng/g) 1–48 h after oral administration in rats (*n* = 6).

Concentration (ng/g)							
	1.0 h	3.0 h	5.0 h	8.0 h	12.0 h	24 h	48 h
**Heart**							
Isoalantolactone	10.1 ± 0.3 ^a^	**44.6 ± 9.5**	21.1 ± 6.5	11.1 ± 1.0	7.2 ± 0.8	-	-
Alantolactone	6.1 ± 1.0	**31.0 ± 16.7**	10.2 ± 2.2	6.9 ± 0.9	6.4 ± 0.3	-	-
**Spleen**							
Isoalantolactone	17.9 ± 7.1	**18.3 ± 8.1**	8.6 ± 4.2	4.4 ± 1.5	-	-	-
Alantolactone	14.8 ± 3.1	**20.0 ± 5.9**	13.1 ± 2.4	-	-	-	-
**Lung**							
Isoalantolactone	26.8 ± 11.5	**83.2 ± 38.1**	21.8 ± 9.2	-	-	-	-
Alantolactone	26.0 ± 10.7	**92.1 ± 45.2**	33.9 ± 5.8	-	-	-	-
**Kidney**							
Isoalantolactone	78.6 ± 20.7	**96.9 ± 9.9**	92.9 ± 11.4	51.4 ± 6.3	49.8 ± 5.1	10.3 ± 4.1	-
Alantolactone	50.8 ± 8.8	**66.6 ± 8.7**	49.2 ± 8.3	35.6 ± 4.9	31.1 ± 4.9	-	-
**Liver**							
Isoalantolactone	170.1 ± 23.8	**311.3 ± 113.5**	243.5 ± 149.4	182.0 ± 33.8	124.3 ± 23.3	20.2 ± 9.8	-
Alantolactone	254.6 ± 24.3	**341.0 ± 24.9**	301.8 ± 69.0	205.4 ± 36.0	167.6 ± 34.5	20.9 ± 6.9	-
**Stomach**							
Isoalantolactone	**1779.7 ± 406.6**	376.5 ± 83.6	219.6 ± 57.8	59.4 ± 27.3	-	-	-
Alantolactone	**1792.7 ± 576.8**	302.0 ± 45.3	210.3 ± 52.0	36.7 ± 11.6	-	-	-
**Small intestine**							
Isoalantolactone	1656.9 ± 821.6	**2461.5 ± 263.7**	1559.6 ± 478.1	614.2 ± 225.4	45.4 ± 9.4	-	-
Alantolactone	1222.1 ± 377.5	**3118.0 ± 389.8**	2033.7 ± 548.0	947.1 ± 420.8	-	-	-
**Colon**							
Isoalantolactone	21.2 ± 2.9	116.4 ± 31.2	147.5 ± 74.7	**486.7 ± 267.1**	455.7 ± 223.9	100.6 ± 27.3	-
Alantolactone	15.6 ± 5.0	167.3 ± 56.5	187.1 ± 89.1	**662.0 ± 273.8**	502.1 ± 164.2	105.2 ± 31.8	-

^a^ Mean ± SD.

### 2.5. Excretion

The excretion of isoalantolactone and alantolactone in bile, urine and feces is illustrated in Table 4, Table 5 and Table 6. After a single oral dose of 90 mg/kg Radix Inulae extract, mean recovery in bile, urine, and feces was 4.18%, 0.044%, and 28.22% for isoalantolactone and 3.33%, 0.024%, and 26.41% for alantolactone, respectively. The process of bile excretion of the lactones did not correlate with the process *in vivo* because rats were restrained when bile was collected. During the experiment, we found that it was difficult to collect the urine and feces samples of each rat in all time points, and while no results were missed in Table 4 and Table 6 no urine and feces were collected for some time periods. This also resulted in that the T_max_ in the urine analysis process seemed insignificant. We collected urine for 48 h to make sure that the total content of lactones in urine was accurate. The extremely variable data suggested the varying degrees of first-pass effect in rats may lead to individual differences which is similar to the phenomenon observed in the pharmacokinetic studies *in vivo* (large standard deviation from the plasma concentration-time profiles in Figure 3). On the other hand, due to the extreme low excretion in urine, total unchanged lactones recovered from all regular excretion routes were still less than 35%. There were very few reported studies about the excretion of sesquiterpene lactones after oral administration in rats, and the results from this study suggested that metabolism was likely to play an important role in their elimination as well. However, further research is required to identify whether poor intestinal absorption or drug metabolism is the main reason that explains the high levels in feces and low concentration *in vivo* after oral administration of lactones.

**Table 4:** Excretion of lactones in rats urine after a single oral administration at 90 mg/kg.

Rat Number	Time (h)							
	0–1	1–3	3–5	5–8	8–12	12–24	24–48	Total (ng)
1								
Isoalantolactone	-	-	92.0	106.8	154.8	58.8	4.8	325.1
Alantolactone	-	-	38.1	32.6	108.4	-	-	179.0
2								
Isoalantolactone	-	639.1	23.2	29.2	51.9	-	-	743.3
Alantolactone	-	250.6	13.0	-	38.5	-	-	301.9
3								
Isoalantolactone	-	28.0	-	102.8	56.3	-	-	187.1
Alantolactone	-	-	-	43.2	13.5	-	-	56.7
4								
Isoalantolactone	32.1	115.0	-	15.0	41.4	31.6	-	235.2
Alantolactone	-	129.3	-	21.4	18.7		-	169.3
5								
Isoalantolactone	-	6.2	-	-	104.3	-	-	110.5
Alantolactone	-	-	-	-	-	49.6	-	49.6
6								
Isoalantolactone	-	-	510.0	554.1	-	-	-	1219.0
Alantolactone	-	-	-	149.7	270.2	-	-	419.9

**Table 5:** Excretion of lactones in rats bile after a single oral administration at 90 mg/kg.

Time (h)	Isoalantolactone (μg)	Alantolactone (μg)
0–1	4.3 ± 2.8 ^a^	4.6 ± 0.7
1–3	12.1 ± 1.1	16.9 ± 1.2
3–5	18.8 ± 5.6	19.3 ± 10.0
5–8	51.4 ± 9.1	61.9 ± 19.2
8–12	77.8 ± 9.7	39.0 ± 7.9
12–15	58.3 ± 12.7	34.6 ± 8.9
15–24	149.9 ± 57.5	69.9 ± 36.8
24–48	81.3 ± 30.3	23.7 ± 4.9
48–72	-	-
Total	453.8	269.8

^a^ Mean ± SD.

**Table 6:** Excretion of lactones in rats feces after a single oral administration at 90 mg/kg.

Rat Number	Time (h)				
	0–8	8–12	12–24	24–48	Total (μg)
1					
Isoalantolactone	-	264.4	5431.4	103.9	5799.7
Alantolactone	-	116.2	3630.1	172.2	3918.5
2					
Isoalantolactone	41.4	1719.7	653.7	146.4	2561.2
Alantolactone	22.0	1263.0	453.9	173.5	1912.3
3					
Isoalantolactone	29.5	20.4	1236.7	77.2	1363.7
Alantolactone	14.1	12.1	952.8	40.5	1019.5
4					
Isoalantolactone	73.7	335.5	731.2	122.7	1263.0
Alantolactone	64.6	256.3	614.6	108.5	1044.0
5					
Isoalantolactone	9.3	1597.9	1110.4	-	2717.6
Alantolactone	5.5	1298.5	910.5	-	2214.5
6					
Isoalantolactone	6.5	35.4	4297.6	289.3	4628.8
Alantolactone	6.5	10.8	2667.2	66.0	2750.5

## 3. Experimental Section

### 3.1. Chemicals and Reagents

Osthole (purity > 98.0%, Figure 1C) as internal standard (IS) was obtained from the National Institute for the Control of Pharmaceutical and Biological Products (Beijing, China). Acetonitrile and methanol, HPLC grade, were purchased from Merck (Darmstadt, Germany). Ultra-pure water for the LC mobile phase was prepared in-house using a Milli-Q system (Millipore, Bedford, MA, USA). All other chemicals were analytical grade and used without further purification.

### 3.2. Apparatus and LC-MS/MS Conditions

Quantification of isoalantolactone and alantolactone was performed on a Waters UPLC system with an Applied Biosystem 5500 QTRAP^®^ hybrid triple-quadrupole mass spectrometer (Applied Biosystems/MDS Sciex, Foster City, CA, USA), equipped with a turbo ion spray source. Chromatographic separation was performed on a ZORBAX Eclipse Plus C18 (50 mm × 2.1 mm, 1.8 μm). Samples were eluted through the column with a gradient of water–formic acid (100:0.5, v/v) and acetonitrile (0 min, 52:48; 4.2 min, 52:48; 4.3 min, 95:5; 5.3 min 95:5; 5.4 min 52:48; 6.4 min 52:48) at a flow rate of 0.4 mL/min at 50 °C. The injection time was 6.4 min.

The mass spectrometer was operated as reported before [19]. Simply, positive mode was selected for the isomers and IS. Quantification was performed using multiple reaction monitoring (MRM) mode of the transitions *m/z* [M+H]^+^ 233.2→187.1 for isoalantolactone and alantolactone and *m/z* [M+H]^+^ 245.0→189.0 for osthole (IS) with DP of 50 V, respectively. The MS parameters were as follows: Ion spray (IS) voltage: 5500 V; temperature: 550 °C; Gas 1 and Gas 2 (nitrogen) were set at 35 and 35 psi, respectively. The curtain gas using nitrogen was set at 40 psi. The optimized collision energies (CE) for isoalantolactone, alantolactone and IS was 19eV. The dwell time was set at 550 ms per transition. MS/MS operating conditions were optimized by infusion of the standard solution (500 ng/mL) of analyte or IS into the ESI source via a syringe pump, respectively. Data acquisition was performed using Analyst 1.5.2 software (Applied Biosystems).

### 3.3. Preparation of Radix Inulae Extract

Radix Inulae (1 kg) was refluxed twice with 95% ethanol (10 L) at 100 °C for 2 h. The extracts were mixed, and ethanol was removed under reduced pressure. The residue was suspended in water and extracted with chloroform. The resulting solution was evaporated under reduced pressure and provided a brown residue that contained isoalantolactone and alantolactone. The two isomeric sesquiterpene lactones in the solution were quantitatively determined by a validated HPLC–UV method [4]. The corresponding concentrations of isoalantolactone and alantolactone in the formulation were 4:3.

### 3.4. Animals, Drug Administration and Sampling

Male Sprague Dawley (SD) rats weighing 200–220 g (7 weeks old) were obtained from the Laboratory Animal Centre of Shanghai Jiao Tong University (Shanghai, China). Animals were acclimated for 7 days to a 12 h light/dark cycle in a temperature-controlled environment with appropriate humidity, in accordance with the National Institutes of Health publication Guide for the Care and Use of Laboratory Animals. All the animal experiments were approved by Shanghai Jiao Tong University Animal Ethics Committee (Shanghai, China).

For a pharmacokinetic study, six male SD rats were oral administrated Radix Inulae extract (90 mg/kg, containing 49.02 mg/kg isoalantolactone and 36.48 mg/kg alantolactone), which was dissolved into a liquid suspension, with concentration of 9 mg/mL using 5% PEG-400. Blood samples (0.3 mL) were obtained from the posterior orbital *venous plexus* to a heparinized tube at 0, 0.16, 0.5, 1, 1.5, 2, 3, 4, 6, 8, 12 and 24 h, respectively, and then centrifuged at 5000 r/min for 15 min. The plasma was collected and stored in −80 °C.

For the tissue distribution study, 42 male SD rats (*n* = 6 for each time point) were selected. After oral administration of Radix Inulae extract (90 mg/kg), heart, liver, lung, spleen, kidney, stomach, colon and small intestine samples were collected at 1, 3, 5, 8, 12, 24 and 48 h, respectively. Time points were formulated according to the concentration–time curves. Before tissue samples were collected, blood-rich tissues like livers and hearts were perfused for the removal of blood, and other organs were washed in saline for three times. Tissue samples were weighed rapidly, rinsed with physiological saline to remove the blood or content, blotted on filter paper, and then stored at −80 °C until analysis.

Urine and feces were collected from six rats after oral administration of 90 mg/kg Radix Inulae extract and each animal was housed in a metabolic cage from 1 to 48 h.

Under light anesthesia with chloral hydrate, bile fistulas in 6 rats were cannulated with PE-5 polyethylene tubing for the collection of bile. After administration, the bile was replaced during 1, 3, 5, 8, 12, 15, 24 and 48 h under freezing. The rats were allowed to recover from anesthesia before receiving a 90 mg/kg oral dose of Radix Inulae extract. Rats were kept in restraining cages during the bile collection period.

### 3.5. Sample Processing

Before analysis, the plasma and urine samples were thawed to room temperature. In a 1.5 mL centrifuge tube, an aliquot of 10 μL of the internal standard working solution (5 ng/mL) was added to 100 μL of collected plasma and urine sample followed by a supplementary addition of 390 μL acetonitrile (4:1, v/v). The tubes were vortexed for 1.0 min. After centrifugation at 14,000 rpm for 10 min, the supernatant was evaporated in a 40 °C water bath by sample concentrator (Nitrogen blowing instrument). The residue was reconstituted with 100 μL mobile phase. After centrifugation at 14,000 rpm for another 5 min, an aliquot of 2 μL supernatant was injected into the LC-MS/MS system.

Each tissue sample was washed with saline and then dried. After weighing (0.05 g for stomach, colon, small intestine and 0.5 g for other tissues), samples were homogenized with 1.5 mL [v (mL)/w (g)] of saline with 10 μL internal standard working solution (25 ng/mL). An aliquot of 1.5 mL tissue homogenate samples was added to an Eppendorf tube and direct precipitation of matrix was performed by adding ice cold acetonitrile (4:1, v/v). The mixture was vortexed on a thermomixer and centrifuged at 14,000 rpm for 20 min at 4 °C. Clear supernatant (8.0 mL) was transferred to a new Eppendorf tube and evaporated to dryness, then the residue was reconstituted with 400 μL mobile phase and transferred to a 96-deep well plate. The final plate was loaded into the autosampler cabinet and 2 μL aliquots were used for LC-MS/MS.

Feces samples were pulverized with a mortar pestle. Five volumes of physiological saline and acetonitrile (1:3, w/v) was added to 0.05 g pulverized feces with IS and vortex mixed for 1.0 min. After centrifugation at 14,000 rpm for 10 min, 10 μL supernatant was diluted with 100 volumes acetonitrile, 2 μL aliquots was injected into the LC-MS/MS system.

Acetonitrile (485 μL) was added in a 5 μL bile sample with 10 μL of the internal standard working solution (25 ng/mL), after centrifugation at 14,000 rpm for 10 min, an aliquot of 2 μL was injected into the LC-MS/MS system.

### 3.6. Preparation of the Standard Solution and Calibration Standard

The stock solution of isoalantolactone, alantolactone and IS (10 mg/mL) was prepared in acetonitrile. The working solutions at various concentrations were obtained by serial dilutions of the stock solution using methanol. All the solutions were stored at 4 °C until analysis. A standard curve calibration was performed at concentrations of 2, 4, 5, 10, 20, 50, 100, 200 and 400 ng/mL by spiking the moderate amounts of working solutions into blank matrix containing plasma, organs bile, urine and feces. After processed as described in 3.5 Sample processing, the samples were injected into the LC-MS/MS system.

### 3.7. Method Validation

The bioanalytical method was validated for selectivity, linearity, low limit of determination (LLOQ), precision, accuracy, recovery and matrix effect according to the principles of Food and Drug Administration (FDA) industry guide [30]. Quality control (QC) samples were prepared at low, medium and high concentrations: 5, 25 and 80 ng/mL for plasma, urine, 1, 10 and 50 μg/mL for bile, 5, 50 and 250 ng/mL for liver and 5, 25, 250 μg/mL for feces, respectively. Accuracy and precision at the LLOQ was also determined for plasma according the the FDA guidance. The preparation process was the same for calibration standards.

## 4. Conclusions

A sensitive, specific and accurate UPLC–MS/MS method was developed for the assessment of isoalantolactone and alantolactone level in rat plasma, heart, liver, spleen, lung, kidney, gastrointestinal, urine, bile and feces with a chromatographic run time of 6.4 min. This method possesses multiple advantages, including high sensitivity with an LLOQ of 2.0 ng/mL for isoalantolactone and 4.0 ng/mL for alantolactone, satisfactory selectivity and simple sample preparation. The analytical procedure was then applied to a pharmacokinetics, tissue distribution and excretion study of isoalantolactone and alantolactone in rats after oral administration of Radix Inulae extract. The results could be useful to understand the mechanism of action of Radix Inulae and provide supporting evidence to guide clinical application of the formulation.

## Supplementary Materials

Supplementary materials can be accessed at: http://www.mdpi.com/1420-3049/20/05/7719/s1.
