# Supplementary material for: Pharmacokinetics, Tissue Distribution and Excretion of Isoalantolactone and Alantolactone in Rats after Oral Administration of Radix Inulae Extract
Source: Molecules. 2015 Apr 28;20(5):7719–36. doi: 10.3390/molecules20057719 (PMC6272429; doi:10.3390/molecules20057719)
Supplement: Supplementary file 1 [file molecules-20-07719-s001.pdf]

Supplmentary

Table S1. Precision, accuracy, extraction recovery and matrix effect for the isoalantolactone and alantolactone in rat plasma, liver, bile, urine and feces (*n* = 18, 6 replicates per day for 3 days).

Sample matrix	Concentration (ng/mL)	RSD (%)		RE (%)		Extraction Recovery (%)	Mean Matrix Effect (%)
		Intra-Day	Inter-Day	Intra-Day	Inter-Day		
Plasma	Isoalantolactone						
	2	3.87	5.98	−7.11	−8.50		
	5	2.84	3.15	−8.17	−8.12	87.11 ± 6.50	103.59 ± 5.90
	25	2.96	3.17	−6.20	5.37	89.13 ± 3.45	101.24 ± 4.59
	80	2.88	4.51	−5.94	7.48	85.30 ± 6.61	99.32 ± 7.12
	Alantolactone						
	4	4.40	5.80	−9.29	−9.58		
	5	3.13	5.35	−10.11	−9.29	83.40 ± 2.33	102.78 ± 3.63
	25	4.20	5.83	2.14	6.13	84.35 ± 4.14	101.58 ± 5.47
	80	3.09	7.11	5.39	3.66	81.76 ± 5.12	100.66 ± 4.35
Liver	Isoalantolactone						
	5	5.39	8.20	−5.79	−11.92	84.20 ± 1.61	105.25 ± 7.09
	50	6.11	9.14	−9.14	−8.46	85.21 ± 3.12	105.51 ± 6.12
	250	5.83	8.69	−9.78	9.53	87.30 ± 4.59	99.31 ± 1.08
	Alantolactone						
	5	6.38	6.20	9.30	9.45	82.22 ± 3.56	104.02 ± 2.58
	50	5.34	8.20	−5.31	−7.56	84.42 ± 3.29	102.78 ± 4.29
	250	5.68	6.88	−4.28	−6.60	86.65 ± 5.47	96.58 ± 4.90
Bile	Isoalantolactone						
	10^3	6.23	6.98	−4.30	−3.78	93.28 ± 5.89	102.66 ± 3.75
	10^4	5.78	7.97	−3.34	5.13	94.20 ± 6.44	95.25 ± 3.31
	5×10^4	7.15	8.66	6.77	6.28	93.60 ± 6.60	105.51 ± 8.47
	Alantolactone						
	10^3	6.99	7.32	−8.60	−5.33	91.24 ± 5.11	104.31 ± 6.63
	10^4	6.53	8.34	−7.71	4.21	92.75 ± 5.33	101.02 ± 6.35
	5×10^4	6.31	8.87	7.38	4.70	90.49 ± 5.54	100.30 ± 3.22
Urine	Isoalantolactone						
	5	4.28	8.13	2.39	13.20	94.99 ± 4.40	105.01 ± 4.48
	25	3.55	7.01	8.64	4.46	88.96 ± 3.88	104.56 ± 4.55
	250	7.34	8.24	4.51	7.57	80.57 ± 4.64	102.23 ± 4.28
	Alantolactone						
	5	3.25	6.99	−7.60	−9.39	93.28 ± 3.97	104.81 ± 4.13
	25	4.57	8.56	6.40	7.30	90.54 ± 5.34	102.24 ± 7.16
	250	8.11	8.79	4.70	6.79	82.23 ± 6.75	100.81 ± 3.99
Feces	Isoalantolactone						
	5×10^3	7.50	7.15	3.61	4.32	91.77 ± 2.62	102.74 ± 2.73
	2.5×10^4	8.76	9.83	4.10	9.52	95.38 ± 2.95	101.79 ± 3.33
	2.5×10^5	4.30	6.67	4.88	4.79	94.15 ± 2.69	104.54 ± 3.72
	Alantolactone						
	5×10^3	8.44	9.56	5.87	6.98	90.01 ± 2.66	102.82 ± 3.59
	2.5×10^4	8.34	8.30	3.60	7.13	93.78 ± 2.43	101.28 ± 1.39
	2.5×10^5	6.10	6.12	3.59	8.03	92.80 ± 2.57	103.46 ± 5.68
